# Comprehensive Analysis of Quantitative Proteomics With DIA Mass Spectrometry and ceRNA Network in Intrahepatic Cholestasis of Pregnancy

**DOI:** 10.3389/fcell.2022.854425

**Published:** 2022-07-22

**Authors:** Dajun Fang, Yan Fang, Weiqiang Zhang, Yun Xiang, Xi Cheng, Mingfeng Liang, Huimin Xia

**Affiliations:** Department of Obstetrics and Gynecology, Guangzhou Women and Children’s Medical Center, Guangdong Provincial Clinical Research Center for Child Health, Guangzhou Medical University, Guangzhou, China

**Keywords:** intrahepatic cholestasis of pregnancy, quantitative proteomics, competing endogenous RNA (ceRNA) network, target therapy, regulatory mechanism

## Abstract

**Background:** Intrahepatic cholestasis of pregnancy (ICP) is a pregnancy-specific complication characterized by pruritus without skin damage and jaundice. The poor perinatal outcomes include fetal distress, preterm birth, and unexpected intrauterine death. However, the mechanism of ICP leading to poor prognosis is still unclear.

**Methods:** We analyzed 10 ICP and 10 normal placental specimens through quantitative proteomics of data-independent acquisition (DIA) to screen and identify differentially expressed proteins. GO, KEGG, COG/KOG, StringDB, InterProScan, Metascape, BioGPS, and NetworkAnalyst databases were used in this study. PITA, miRanda, TargetScan, starBase, and LncBase Predicted *v.2* were used for constructing a competing endogenous RNA (ceRNA) network. Cytoscape was used for drawing regulatory networks, and cytoHubba was used for screening core nodes. The ICP rat models were used to validate the pathological mechanism.

**Results:** GO, KEGG, and COG/KOG functional enrichment analysis results showed the differentially expressed proteins participated in autophagy, autophagosome formation, cofactor binding, JAK-STAT signaling pathway, and coenzyme transport and metabolism. DisGeNET analysis showed that these differentially expressed proteins were associated with red blood cell disorder and slow progression. We further analyzed first 12 proteins in the upregulated and downregulated differentially expressed proteins and incorporated clinicopathologic parameters. Our results showed HBG1, SPI1, HBG2, HBE1, FOXK1, KRT72, SLC13A3, MBD2, SP9, GPLD1, MYH7, and BLOC1S1 were associated with ICP development. ceRNA network analysis showed that MBD2, SPI1, FOXK1, and SLC13A3 were regulated by multiple miRNAs and lncRNAs.

**Conclusion:** ICP was associated with autophagy. The ceRNA network of MBD2, SPI1, FOXK1, and SLC13A3 was involved in ICP progression, and these core proteins might be potential target.

## 1 Introduction

Intrahepatic cholestasis of pregnancy (ICP), a pregnancy-specific disease is characterized by maternal pruritus and dysregulation of hepatic functions during the third trimester (Mashburn et al., 2021). An elevated level in serum bile acids resulted in many adverse outcomes, such as preterm labor, fetus intrauterine hypoxia, meconium-stained amniotic fluid (Dixon and Williamson, 2016). The risk of stillbirth was increased significantly when the serum bile acid concentration was greater than 40 umol/L (Ovadia et al., 2019). The incidence rate varies among different ethnic groups and was less than 2% a year (Walker et al., 2020). Therapies for ICP were largely empirical. Ursodeoxycholic acid (UDCA), as the first-line treatment for ICP, obviously relieved itching symptoms and improved biochemical indexes. However, results of the largest randomized placebo-controlled study showed UDCA did not reduce the incidence of perinatal poor prognosis compared with placebo (Roediger and Fleckenstein, 2021). At present, few studies on the mechanism of ICP development existed, so exploring the core molecules of adverse pregnancy outcomes caused by ICP was very important.

The noncoding transcripts with a length of at least 200-nt were defined as long noncoding RNAs (lncRNAs) (Bridges et al., 2021). lncRNAs played an important role in a variety of biological processes, such as cancer progression (Qi et al., 2021), inflammatory bowel disease (Jiang and Zhang, 2021), cell autophagy in breast cancer (Li et al., 2021a), and energy metabolism (Tan et al., 2021). Zhou and his colleagues noted that serum expression levels of three lncRNAs—ENST00000505175.1, ASO3480, and ENST00000449605.1 had diagnostic value for ICP patients (Zou et al., 2021). Hu et al. (2018) indicated that there was an elevated expression of Linc02527 in the placenta and serum of ICP patients, and functional studies showed that Linc02527 enhanced the autophagy and proliferated the ability of HTR8 cells. However, the study on how lncRNAs of the placenta influenced the fetal development and placental function was still rare.

miRNAs were noncoding RNAs composed of 21–24 nucleotides and contained broad regulatory functions, such as cell cycle, autophagy, metabolism, immune, and cell death (Li et al., 2021b). Some studies showed the exosomes in urinary samples of ICP patients had high expression levels of miR-21, miR-29a, and miR-590-3p which together inhibited the ICAM expression correspondingly, leading to increasing ICP incidence (Jiang et al., 2019); moreover, some researchers constructed a binary logistic regression model through analyzing miR-151-3p, miR-300, miR-671-3p, and miR-369-5p expressions in urine and found that the combination of these four miRNAs had a good diagnostic value for ICP (Ma et al., 2016). The elevated expressions of miR-371a-5p, miR-6865-5p, and miR-1182 in the serum of ICP patients were positively associated with total bile acids, and they might be noninvasive biomarkers (Zou et al., 2018). Zhang et al. (2014) demonstrated that the upregulated miR-148 expression in the placenta might be related to immune response in the pathological process of ICP through regulating the human leukocyte antigen G (HLA-G) expression. Furthermore, miR-148a might be involved in estradiol-induced ICP development *via* influencing the PXR signal pathway and MRP3 expression (Rao et al., 2017). Although there were some research studies about miRNAs in ICP, the mechanism of miRNA regulatory network was few.

In this study, we analyzed the protein expression in the placenta of ICP and normal pregnancies *via* quantitative proteomics of data-independent acquisition (DIA). Our results showed that compared with the normal placenta, the differentially expressed proteins in ICP were involved in autophagosome regulation. Among the top 24 differentially expressed proteins, HBG1, SPI1, HBG2, HBE1, FOXK1, KRT72, SLC13A3, MBD2, SP9, GPLD1, MYH7, and BLOC1S1 were associated with ICP development significantly; furthermore, MBD2, SPI1, FOXK1, and SLC13A3 were regulated by multiple miRNAs and lncRNAs. On the whole, our study on ICP might provide potential drug targets to improve the adverse pregnancy outcomes of ICP patients.

## Materials and Methods

### Placental Specimens

In this study, we collected placenta samples and clinical data from 10 normal pregnancy patients and 10 ICP patients who underwent cesarean or vaginal delivery. The diagnostic criteria of ICP were gestational pruritus, and the concentration of serum bile acids was greater than 10 umol/L, and the details of all patients are shown in the supplementary file (Table 1). All samples were obtained with informed patients’ consent before collection and approved by the Guangzhou Women and Children’ Medical Center Ethics Committee. Moreover, the collected samples were fixed in formalin or maintained at −196°C (liquid nitrogen) for further study.

**TABLE 1:** Top 30 nodes ranked by the Degree method.

Rank	Name	Score	Log2FC	*p*-value	Regulated
1	NEDD8	8	−0.341	0.039	Down
2	ATG5	6	−0.347	0.022	Down
2	FECH	6	0.382	0.035	Up
4	ACAT2	5	0.575	0.001	Up
4	HBE1	5	0.952	0.019	Up
6	ACOX3	4	−0.287	0.049	Down
6	CTPS2	4	0.332	0.032	Up
6	GABARAPL2	4	0.465	0.004	Up
6	GABARAPL1	4	−0.278	0.028	Down
6	MLST8	4	−0.494	0.001	Down
6	PLEK	4	0.363	0.049	Up
6	RANBP6	4	0.483	0.006	Up
6	HMBS	4	0.685	0.013	Up
6	PRKAG2	4	−0.362	0.042	Down
6	SDC1	4	−0.541	0.01	Down
16	PBDC1	3	−0.293	0.037	Down
16	ECI2	3	−0.448	0.001	Down
16	ACE	3	−0.431	0.034	Down
16	PEX14	3	−0.467	0.027	Down
16	GNG10	3	0.531	0.035	Up
16	ADSS1	3	0.548	0.027	Up
16	PSAT1	3	1.767	0.01	Up
16	WIPI1	3	−0.308	0.008	Down
16	RICTOR	3	−0.289	0.026	Down
16	UROD	3	0.419	0.031	Up
16	PAK1	3	−0.272	0.037	Down
16	CREBBP	3	−0.397	0.048	Down
16	SMARCB1	3	−0.623	0.021	Down
16	HBG1	3	1.554	0.003	Up
16	HBM	3	1.111	0.011	Up

### Quantitative Proteomics of Data-Independent Acquisition

Quantitative proteomics of data-independent acquisition (DIA) integrated “shotgun” and a technique of selected reaction monitoring (SRM), also known as multiple reaction monitoring (MRM), and it performed quantitative and qualitative assessments based on mass-to-charge (m/z). The collected placental specimens underwent a series of protein extraction, protease dissociation, database establishment, mass spectrum, DIA, quality control, and protein identification steps, and we obtained proteins of samples, according to the detected spectrum counting and peak. The mass spectrometry data were collected using a Q Exactive Plus mass spectrometer in tandem EASY-nLC 1,200 liquid phase. The details of the parameters are as follows: analysis column (50 um*15 cm, C18, 2 um, 100 Å), mobile phase (A: 0.1% formic acid, B: 0.1% formic acid and 80% ACN), velocity (300 nL/min), MS1 (R = 70K, AGC = 3e6, Max IT = 30 ms, scan range = 350–1,250 m/z), MS2 (R = 17.5 K, AGC = 1e6, Max IT = 50 ms), and collisional energy (value = 28). The differentially expressed proteins were identified according to the fold change (FC) of the protein expression (FC > 1.2 or FC < 0.83, *p* < 0.05).

### Orthogonal Projections to Latent Structure Discriminant Analysis

OPLS-DA were powerful statistical modeling tools (Worley and Powers, 2016). In this study, it was used to provide insights into separations between the ICP group and NC group based on high-dimensional spectral measurements.

### Enrichment Analysis

In order to study the function of proteins, the identified proteins were annotated in the functional database. BLAST comparison (blastp, evalue ≤ 1e-5) was used to annotate GO, KEGG, and COG databases, and the comparison result with the highest score was selected as the annotation result. BLAST software combined with UniProt and InterProScan databases was used for domain annotation. The variation trend of differential proteins was evaluated by a volcano map and heatmap display analysis. The GO items, KEGG pathway, and COG functions of the differential proteins were performed to determine functional enrichment analysis. Metascape was used to further verify the summary of hub protein enrichment analysis in PaGenBase, DisGeNET, and Cell Type Signature.

### STRING

STRING is a database used to predict protein–protein interactions (PPI) including direct (physical) and indirect (functional) associations (Szklarczyk et al., 2021); If there were no corresponding species in the database, the sequences of proximal species were extracted. Then, blast comparison was performed between the differential protein sequences to obtain the corresponding interaction information and construct the network. In this study, we matched PPI data in the human database, and STRING in this study was used for exploring the interactions within differentially expressed proteins of ICP placenta.

### Cytoscape

Cytoscape Web was an interactive tool, and it visualized protein interactions (Shannon et al., 2003). In this study, Cytoscape was used to visualize the PPI network from STRING and classify proteins according to their expression levels. Moreover, Cytoscape was also used to construct a competing endogenous RNA (ceRNA) network. CytoHubba, a plugin of Cytoscape, was used to rank proteins according to degree algorithms. Plugin-MCODE was used to explore chief sub-PPI networks.

### BioGPS

BioGPS was a free extensible and customizable gene annotation portal (Wu et al., 2016). In this study, BioGPS was used to study the function and position of core proteins.

### NetworkAnalyst

NetworkAnalyst was a visual analytics platform for gene regulation, gene co-expression, and generic and drug–gene interaction networks (Zhou et al., 2019). NetworkAnalyst in our study was used for exploring transcription factors that regulated core proteins.

### ceRNA Network Construction

In this study, PITA, miRanda, and TargetScan databases were used to identify the upstream miRNAs of core proteins. StarBase and LncBase Predicted *v.2* were used to find the upstream lncRNAs of miRNAs. Furthermore, the results were taken, the intersection and network were constructed by Cytoscape.

### Animal and Placental Explant Models

The 18 female and 18 male Sprague–Dawley rats (body weight: 250 ± 20 g) were purchased from Guangzhou Ruige Biotechnology Co., LTD. The animals were given access to food and water. One female rat was mated with one male overnight, and the day when a vaginal plug was found was defined as day 0. The ICP rat model was constructed as follows: from day 13, the rats were injected intraperitoneally with estradiol benzoate (5 mg/kg, MCE, China) until day 20, which we named it the ICP group. The NC group was treated with equal DMSO consecutively. The pregnant rats were sacrificed, and the placenta was harvested in day 21. All experimental operations were approved by the animal ethics committee of Guangzhou Women and Children’ Medical Center Ethics Committee. As for placental explant models, the placenta was obtained from normal pregnancy patients, and it was shredded and stimulated for 48 h with 100 uM cholid acid (MCE, China) to construct the ICP model.

### R Packages and Western Blotting

R packages including pheatmap, clusterProfiler, and igraph were used in this work. The antibodies used in Western blotting are as follows: actin (GB12001; 1:3000; Servicebio, China), LC3 (14600-1-AP; 1:1,000; Proteintech, United States), and Beclin 1(GB112053; 1:1,000; Servicebio, China). The AlphaView tool was used to analyze the optical density values of the target band.

### Statistics Analysis

Results expressed as mean ± standard error (m ± SE) were analyzed by two-tailed Student’s t-test. Fisher’s test was used for clinical data table. The statistical significance was defined as *p* < 0.05 by GraphPad Prism software (version 6.0, GraphPad Software, United States).

## Results

### Functional Enrichment Analysis of Differentially Expressed Proteins Based on Quantitative Proteomics

**FIGURE 1:**
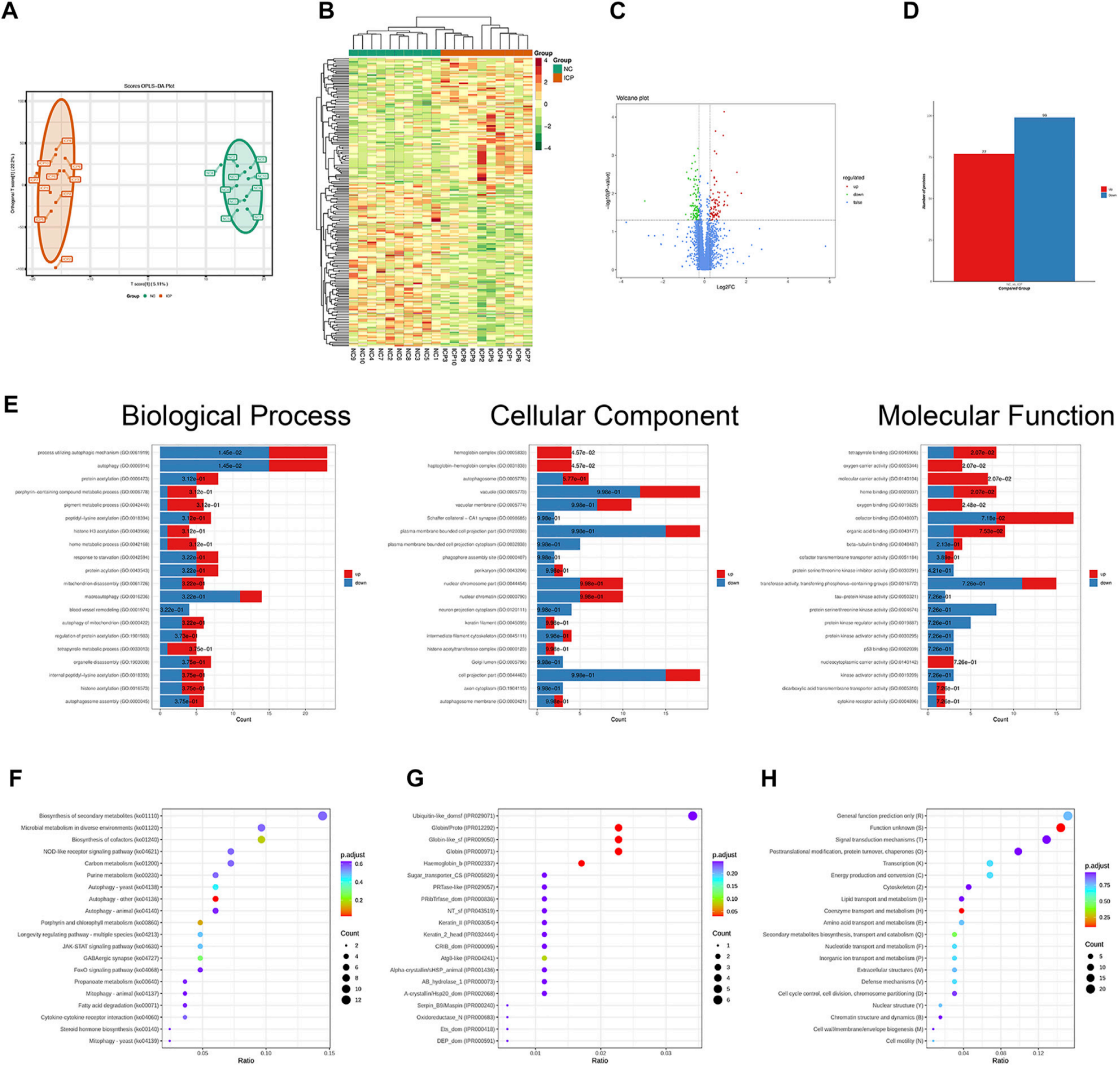
Functional enrichment analysis of differentially expressed proteins based on quantitative proteomics. **(A)** Results of OPLS-DA analysis. **(B)** Heatmap of ICP differentially expressed proteins. **(C)** Volcano map of ICP differentially expressed proteins. **(D)** Histogram about the number of differentially expressed proteins. **(E)** GO analysis of ICP differentially expressed proteins. **(F)** KEGG pathway analysis of ICP differentially expressed proteins. **(G)** Domain enrichment analysis of ICP differentially expressed proteins. **(H)** KOG enrichment analysis of ICP differentially expressed proteins.

We collected placentas from 10 normal pregnancies and 10 ICP patients. In this study, OPLS-DA was an alternative method of principal component analysis. The results of OPLS-DA analysis areshown (Figure 1A). Based on the quantitative proteomics of DIA and the standard of significance (FC > 1.2 or FC < 0.83, *p* < 0.05), we found 77 upregulated proteins and 99 downregulated proteins which were rendered as visual heatmap (Figure 1B), volcano map (Figure 1C), and histogram (Figure 1D). GO enrichment analysis showed that the differentially expressed proteins were implicated in a wide range of biological processes, such as the process utilizing the autophagic mechanism, autophagy, protein acetylation, porphyrin-containing compound metabolic process, and pigment metabolic process. The proteins that participated in the cellular component contained hemoglobin complex, haptoglobin–hemoglobin complex, autophagosome, vacuole, vacuolar membrane, and Schaffer collateral-CA1 synapse. The molecular functions were tetrapyrrole binding, oxygen carrier activity, molecular carrier activity, organic acid binding, and protein serine/threonine kinase inhibitor activity, etc. (Figure 1E). KEGG pathway analysis demonstrated that these proteins were involved in biosynthesis of second metabolites, microbial metabolism in the diverse environment, biosynthesis of cofactors, NOD-like receptor signaling pathway, autophagy, and carbon metabolism. (Figure 1F). Domain enrichment analysis revealed that Atg8-like, Ubiquitin-like_domsf, Globin/Proto, Keratin_II, Serpin_B9/Maspin, and Oxidoreductase_N were included (Figure 1G). KOG enrichment analysis showed that signal transduction mechanisms (T), lipid transport and metabolism (I), transcription (K), energy production and conversion (C), and posttranslational modification, protein turnover, and chaperones (O) were covered (Figure 1H).

### Selection of Core Proteins From the Prediction of the Protein–Protein Interaction Network

**FIGURE 2:**
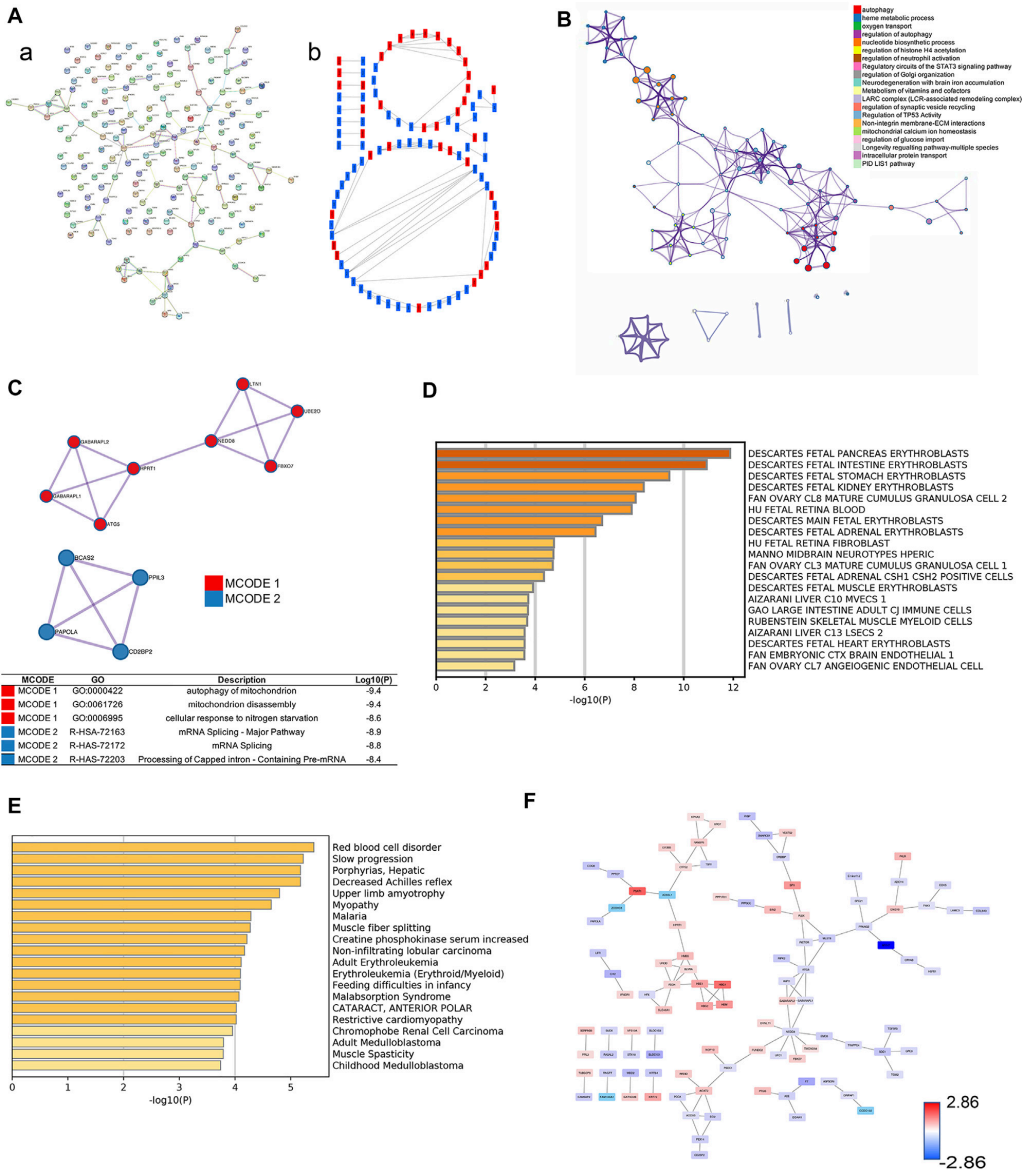
Selection of core proteins from the PPI network. **(A)** PPI network of ICP differentially expressed proteins. **(A)** Results from the STRING database. **(B)** Results from the Cytoscape database. Red color: upregulated proteins; blue color: downregulated proteins. **(B)** Network about enriched terms of ICP differentially expressed proteins in the Metascape database (color by cluster ID). **(C)** MCODE analysis of ICP differentially expressed proteins and functional enrichment analysis of proteins in MCODE 1/2. **(D)** Summary of enrichment analysis in Cell Type Signature. **(E)** Summary of enrichment analysis in DisGeNET. **(F)** PPI network by value of protein expression difference.

The PPI network of differentially expressed proteins was made by using the STRING database and Cytoscape (Figure 2A). To further capture the relationships between proteins, Metascape was used in this study for enrichment analysis and enriched term network construction, where terms with a similarity >0.3 are connected by edges. The results displayed that the core terms included autophagy, regulation of histone H4 acetylation, regulation of TP53 activity, nucleotide biosynthetic process, and oxygen transport (Figure 2B). The proteins in the PPI network were ranked by the degree algorithm, and the results showed top 20 proteins were NEDD8, ATG5, FECH, ACAT2, HBE1, ACOX3, CTPS2, GABARAPL2, GABARAPL1, MLST8, PLEK, RANBP6, HMBS, PRKAG2, SDC1, PBDC1, ECI2, ACE, PEX14, and GNG10 (Table 1). The MCODE algorithm was applied to identify densely connected network components and showed that FBXO7, LTN1, UBE2O, NEDD8, HPRT1, GANARAPL1, GABARAPL2, and ATG5 were included in MCODE1, and CD2BP2, PPIL3, PAPOLA, and BCS2 were components of MCODE2. Furthermore, the proteins in MCODE 1 were associated with autophagy of the mitochondrion, mitochondrion disassembly, and cellular response to nitrogen starvation. The proteins in MCODE 2 were connected with the mRNA splicing-major pathway, mRNA splicing, and processing of capped intron-containing pre-mRNA (Figure 2C).

**FIGURE 3:**
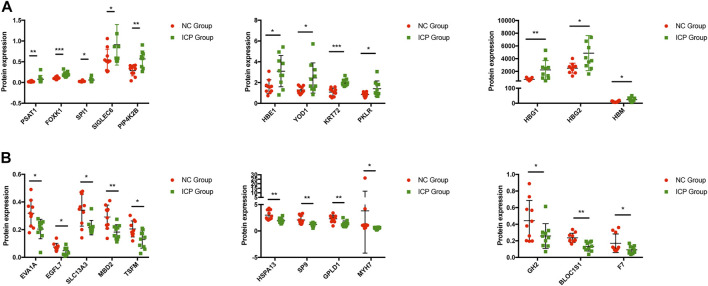
Expression of core proteins. **(A)** Expression of upregulated proteins. **(B)** Expression of downregulated proteins. Error bars, SD. *p < 0.05; **p < 0.01; ***p < 0.001.

The summary of enrichment analysis in the cell type signature showed that Descartes fetal pancreas, intestine, stomach, and kidney erythroblasts were included (Figure 2D). Summary of enrichment analysis in DisGeNET showed that red blood cell disorder, slow progression, upper limb amyotrophy, myopathy, and feeding difficulties in infancy, etc., were included (Figure 2E). To seek core proteins, we ranked the proteins according to the value of expression differences (Figure 2F), and our results showed that the top 12 upregulated proteins were PSAT1, HBG1, HBM, SPI1, PIP4K2B, HBG2, HBE1, FOXK1, YOD1, KRT72, PKLR, and SIGLEC6 (Figure 3A). The top 12 downregulated proteins were HSPA13, EVA1A, EGFL7, SLC13A3, MBD2, TSFM, SP9, GPLD1, GH2, BLOC1S1, F7, and MYH7 (Figure 3B).

### Clinical Significance of Core Proteins

**FIGURE 4:**
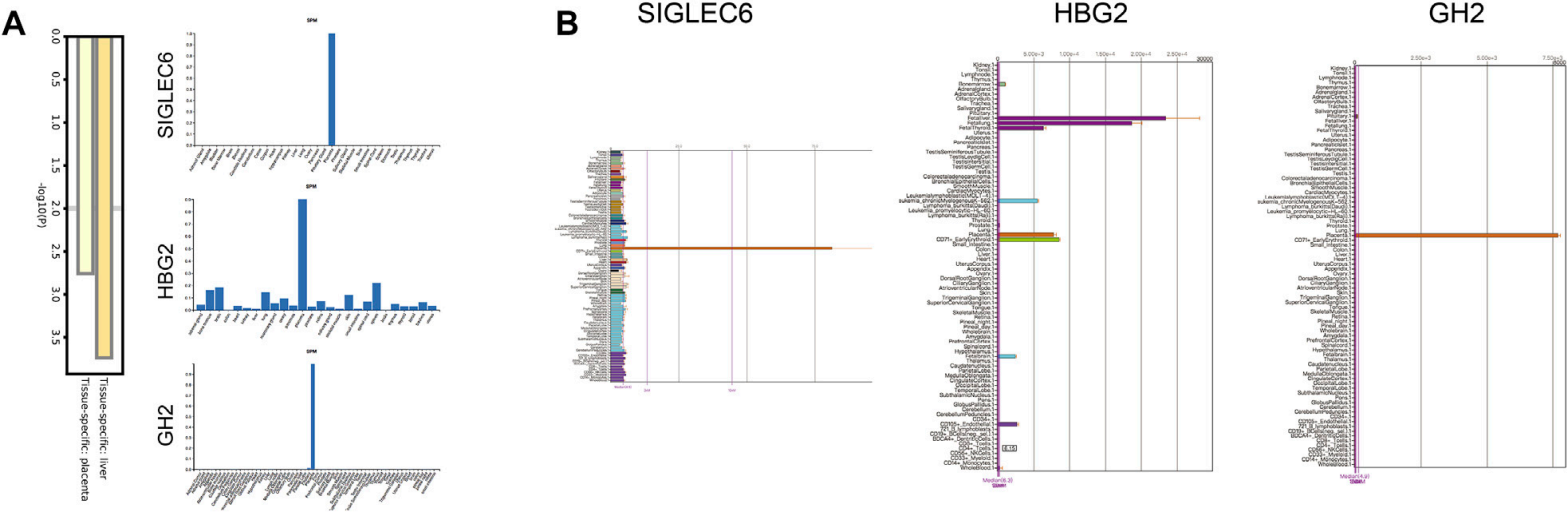
Tissue specificity of the protein expression. **(A)** Summary of enrichment analysis in PaGenBase. **(B)** Expression positioning of SIGLEC6, HBG2, and GH2 in the BioGPS database.

We further performed the enrichment analysis in PaGenBase, and our results showed that most core proteins had tissue-specific expression patterns, especially in the liver and placenta. SIGLEC6, HBG2, and GH2 were highly expressed in the placenta predominantly among core proteins (Figure 4A). The tissue-specific results were also verified by the BioGPS database (Figure 4B). Furthermore, we collected clinical data on patients, including age, delivery gestational age (week), fetal weight (g), and expression of core proteins. Our results showed that ICP development was closely associated with delivery gestational age, and HBG1, SPI1, HBG2, HBE1, FOXK1, KRT72, SLC13A3, MBD2, SP9, GPLD1, MYH7, and BLOC1S1 were linked to the occurrence of ICP. Moreover, the KRT72 expression remained very low in normal human pregnancy and extremely high in ICP patients (Table 2).

**TABLE 2:** Correlation between intrahepatic cholestasis of pregnancy and clinicopathologic parameters.


Parameter	N (%)	Intrahepatic cholestasis of pregnancy		
		NC Group	ICP Group	*p*
Age (years)				0.6563
<30	10 (50)	4	6	
≥30	10 (50)	6	4	
Delivery gestational age (week)				0.0230*
<39 + 2	10 (50)	2	8	
≥39 + 2	10 (50)	8	2	
Fetal weight (g)				0.1789
<3100	10 (50)	3	7	
≥3100	10 (50)	7	3	
PSAT1; HBM; PIP4K2B; YOD1; PKLR; SIGLEC6				0.1789
High expression	10 (50)	3	7	
Low expression	10 (50)	7	3	
HBG1; SPI1; HBG2; HBE1				0.0230*
High expression	10 (50)	2	8	
Low expression	10 (50)	8	2	
FOXK1				0.0011**
High expression	10 (50)	1	9	
Low expression	10 (50)	9	1	
KRT72				<0.0001***
High expression	10 (50)	0	10	
Low expression	10 (50)	10	0	
HSPA13; EVA1A; EGFL7				0.1789
High expression	10 (50)	7	3	
Low expression	10 (50)	3	7	
SLC13A3; MBD2; SP9; GPLD1; MYH7				0.0230*
High expression	10 (50)	8	2	
Low expression	10 (50)	2	8	
TSFM; GH2; F7				0.6563
High expression	10 (50)	6	4	
Low expression	10 (50)	4	6	
BLOC1S1				0.0011**
High expression	10 (50)	9	1	
Low expression	10 (50)	1	9	

The bold values are intended to highlight statistically significant *p* values.

### ceRNA and Transcription Factor Regulatory Network of 12 Clinically Significant Proteins

**FIGURE 5:**
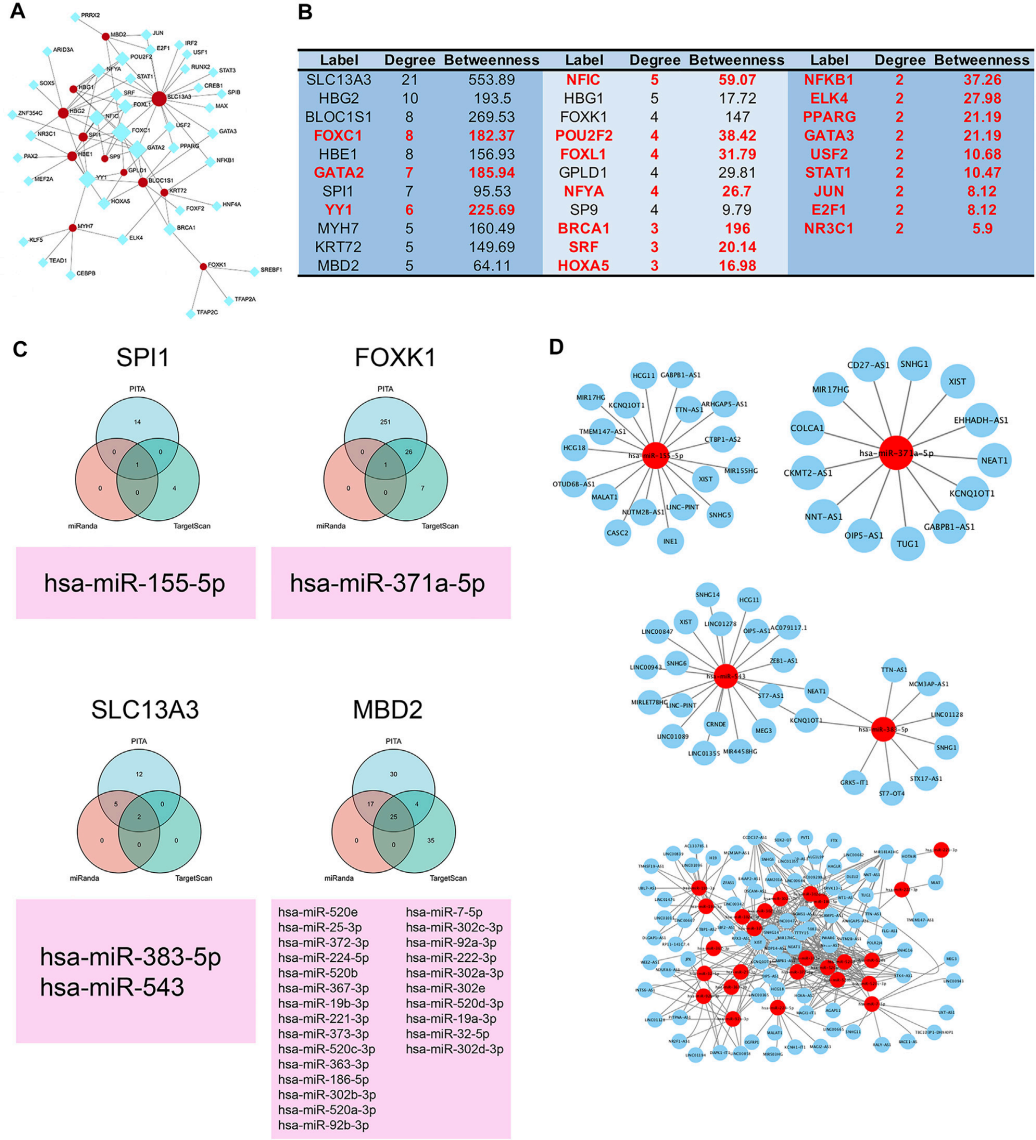
ceRNA and transcription factor regulatory network of 12 clinically significant proteins. **(A)** Transcription factor regulatory network of 12 clinically significant proteins. **(B)** Table of transcription factor regulatory network and 12 clinically significant proteins ranked by degree and betweenness. **(C)** Three independent miRNA target databases were used to predict the potential miRNAs for SPI1, FOXK1, SLC13A3, and MBD2. **(D)** lncRNA–miRNA network.

To explore the regulatory mechanism, we constructed the transcription factor-protein network. Our results showed HBG1, SPI1, HBE1, SP9, GPLD1, BLOC1S1, KRT72, and SLC13A3 were regulated by FOXC1, and HBG1, HBG2, SPI1, SP9, BLOC1S1, KRT72 and SLC13A3 were regulated by GATA2. Moreover, YY1 regulated six protein expressions, and transcription factor NFIC regulated five protein expressions (Figures 5A,B). In addition, combined with PITA, miRanda, and TargetScan databases, we found hsa-miR-155-5p regulated the SPI1 expression, hsa-miR-371a-5p was on the upstream of FOXK1, hsa-miR-383-5p and hsa-miR-543 regulated the SLC13A3 expression, and hsa-miR-520e. was on the upstream of MBD2 (Figure 5C). No miRNAs were found to target HBG1, HBG2, HBE1, KRT72, SP9, GPLD1, MYH7, and BLOC1S1. Moreover, we further studied the lncRNA on the upstream of miRNAs based on Starbase and LncBase Predicted *v.2* database. MIR17HG, HCG18, MALAT1, INE1, CASC2, and SNHG5 were involved in the regulation of hsa-miR-155-5p. NEAT1, XIST, SNHG1, CKMT2-AS1, COLCA1, and TUG1 regulated hsa-371a-5p expression. NEAT1 and KCNQ1OT1 were coregulatory factor of hsa-miR-543 and hsa-miR-383-5p. As for MBD2, the targeted ceRNA network was very complex, and a variety of miRNAs and lncRNAs participated in the regulation of MBD2. KCNQ1OT1 and XIST were common regulatory molecules of all four proteins (Figure 5D).

### Validation of *In Vitro* and *In Vivo* Experimental Models

**FIGURE 6:**
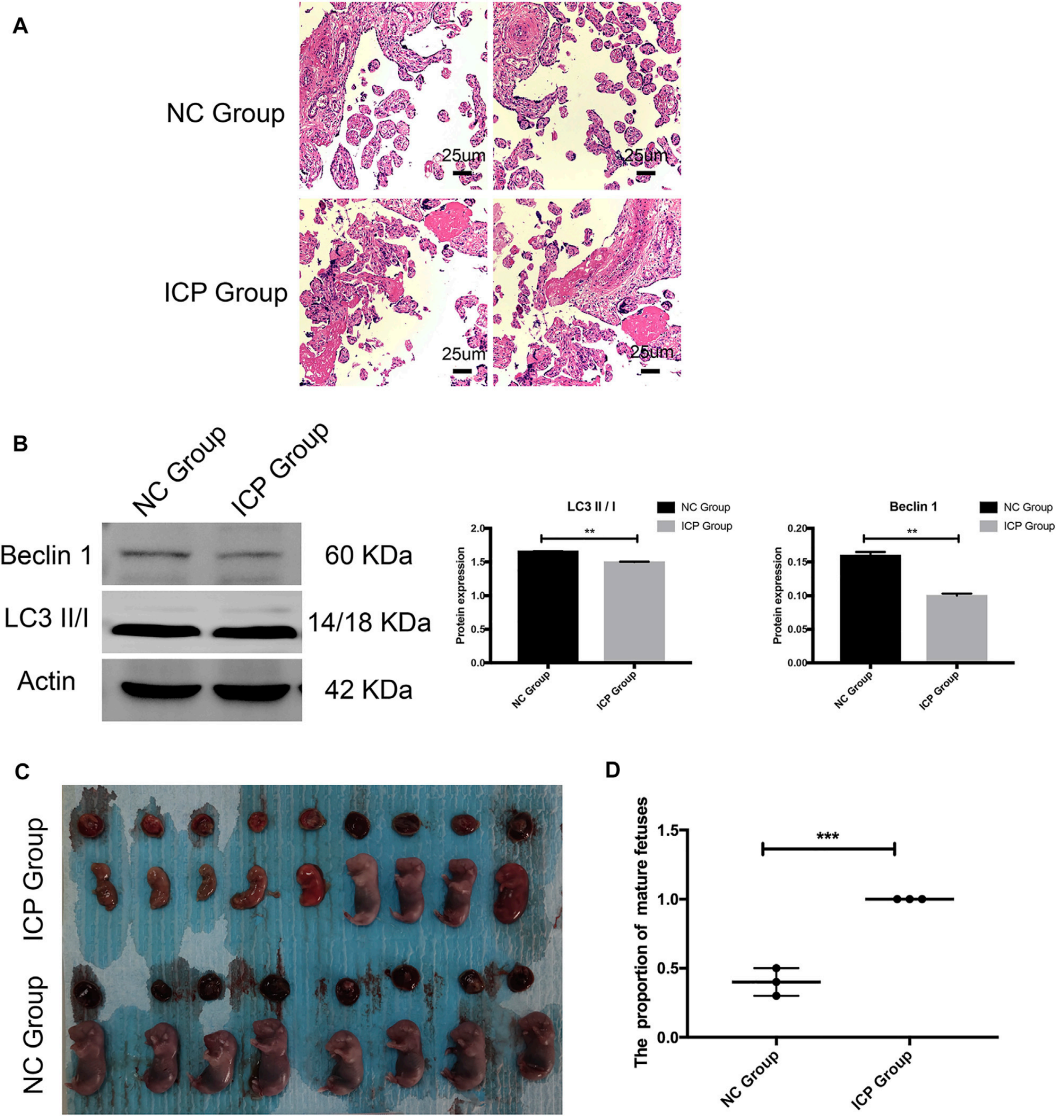
Validation of *in vitro* and *in vivo* experimental models. **(A)** HE results of the placental explant. NC Group: normal placenta; ICP group: normal placenta was stimulated for 48 h with 100 uM cholid acid. **(B)** Western blotting results and gray histogram of the placental explant. NC group: normal placenta; ICP group: the normal placenta was stimulated for 48 h with 100 uM cholid acid. **(C)**Appearance of fetal and placenta in ICP and normal pregnancy rat models. **(D)** Proportion of mature fetuses in ICP and normal pregnancy rat models. SD. *p < 0.05; **p < 0.01; ***p < 0.001.

The placental explant from normal placenta was stimulated for 48 h with 100 uM cholid acid. HE staining results showed that compared with the NC group, the placenta in the ICP group had narrower intervillous space and increased fibrinous necrosis of placental villi (Figure 6A). The Western blotting results of the explant demonstrated that compared with the NC group, the expressions of autophagy-related proteins—Beclin 1 and LC3 II/1 were reduced significantly, which was consistent with our quantitative proteomics (Figure 6B). ICP animal models demonstrated immature fetal rats and placentas were more common, and most fetal rats were not viable *in vitro*. However, in the NC group, all fetal rats developed well (Figures 6C–6D).

## Discussion

DIA had the advantages of panoramic scanning, high data utilization rate, high repeatability, high quantitative accuracy, and data traceability compared with data-dependent acquisition (DDA) (Martinez-Val et al., 2021). In this study, through comprehensive analysis of quantitative proteomics data based on the DIA technique, we screened the different proteins from placental specimens. Enrichment analysis based on a wide variety of tools (GO, KEGG, COG, UniProt, InterProScan, and Metascape) was used in our research to summarize the functional characteristics of different proteins (Fabris et al., 2020). The results of enrichment analysis showed that the differently expressed proteins and core MCODE of the PPI network were involved in autophagy, autophagosome formation, and metabolism regulation. Further analysis of clinical information showed that HBG1, SPI1, HBG2, HBE1, FOXK1, KRT72, SLC13A3, MBD2, SP9, GPLD1, MYH7, and BLOC1S1 were linked to the occurrence of ICP. Among these proteins, SPI1, FOXK1, SLC13A3, and MBD2 were regulated by the ceRNA network. *In vitro* and *in vivo* experimental models also verified that impaired fetal and placental developments were present in the ICP group. Altogether, our results might provide potential targets for ICP therapy to improve the adverse pregnancy outcomes.

KCNQ1OT1 and XIST, as a common regulatory molecule of SPI1, FOXK1, SLC13A3, and MBD2 played in a vital role in multiple pregnancy-related disease. KCNQ1OT1 was one of the mammalian fetal growth-associated imprinted genes. Some studies showed that downregulated KCNQ1OT1 in the placenta was related to the selective intrauterine growth restriction of monozygotic twins (Gou et al., 2017), Beckwith-Wiedemann syndrome (BWS), and Silver-Russell syndrome (SRS) (Azzi et al., 2014; Giabicani et al., 2017). XIST was ∼19 kb lncRNA which controlled the inactivation of the entire X chromosome in female placental mammals (Lu et al., 2020). Li’s study showed XIST was downregulated in gestational diabetes mellitus (GDM) and could serve as a diagnostic biomarker. The XIST/miR-497-5p/FOXO1 pathway reduced the cell viability of trophoblast cells caused by high glucose (Li et al., 2021c). Moreover, XIST had a dramatic decline in the expression when primordial follicle pool was formed in fetal development, and the downregulated XIST led to more miR-23b-3p/miR-29a-3p expressions by preventing the export of pre-miR-23b/pre-miR-29a to the cytoplasm. XIST overexpression resulted in an increased STX17-target of miRNA expression, which in turn accelerated oocyte death (Zhou et al., 2021).

In our study, the enrichment pathway was closely related to autophagy. Autophagy is classified as microautophagy, chaperone-mediated autophagy, and macroautophagy (Zhong et al., 2016). It was a process in which cytoplasmic proteins or organelles were phagocytosed into vesicles and fused with lysosomes to form autophagolyosomes and degrade their encapsulated contents, thus fulfilling the metabolic needs of the cell itself and renewing some organelles (Li et al., 2020). The pathway that regulated autophagy included the PI3K complex, AMPK, p-ERK, mTOR, and P53, etc. (Cao et al., 2021). Autophagy played a crucial role in fetal and placental developments. Many pregnancy-related diseases, such as preeclampsia, ICP, intrauterine growth restriction (IUGR), preterm delivery, and GDM were related to autophagy (Kasture et al., 2021). Furthermore, many biological processes during pregnancy were associated with autophagy, such as fertilization and embryogenesis, implantation, the function of trophoblast, decidualization, maintenance of pregnancy, and immune crosstalk at the maternal–fetal interface (Zhao et al., 2020). As for autophagy in ICP, Shan’s understanding was that defects in autophagy were the basis of pathogenesis. Bile acids inhibited autophagy by binding to Farnesoid X receptor (FXR) to activate the mTOR signaling pathway, impair autophagy-promoting gene PPARα expression, and suppress the autophago-lysosome fusion process (Shan et al., 2021). However, Hu’s study showed that autophagy was activated in ICP *via* Linc02527—miR-3185–AT5/ATG7 signaling pathway (Hu et al., 2018). Obviously, this idea was contradictory to our research and views of Shan. The paradox might be due to heterogeneity spatially and temporally in placental sampling of ICP or other restrictions.

## Conclusion

In summary, our study was the first to analyze the protein profiles with 10 pairs of the ICP placenta comprehensively and construct the ceRNA network. The enrichment analysis showed the destroyed autophagy in the ICP placenta. The core proteins – SPI1, FOXK1, SLC13A3, and MBD2, and their upstream lncRNAs and miRNAs might be potential drug targets to alleviate perinatal adverse outcomes in ICP patients. KCNQ1OT1 and XIST, as a common regulatory lncRNA might play a vital role in ICP development, and it was necessary for us to further explore their functions. Moreover, the drugs targeting autophagy-related proteins might be a promising future for ICP treatment.

## Data Availability

The datasets presented in this study can be found in online repositories. The names of the repository/repositories and accession number(s) can be found below: ProteomeXchange Consortium via the iProX partner repository with the dataset identifier PXD031472 https://www.iprox.cn/page/project.html?id=IPX0004025000 in iProX database.
